# Loss of serotonergic function in carriers of *PRKN* mutations: a [^11^C]DASB PET study

**DOI:** 10.1007/s00259-025-07693-2

**Published:** 2026-01-29

**Authors:** Edoardo Rosario de Natale, Heather Wilson, Joji P. Verghese, Eoin Mulroy, Savvas Antoniadis, Alana Terry, Francesco Cavallieri, Micol Avenali, Pasquale Nigro, Varvara Valotassiou, Eugenii A. Rabiner, Stephen Mullin, Nicola Tambasco, Maria Teresa Pellecchia, Georgia Xiromerisiou, Vicky L. Marshall, Esther Sammler, Enza Maria Valente, Franco Valzania, Kailash P. Bhatia, Marios Politis

**Affiliations:** 1 Neurodegeneration Imaging Group, University of Exeter Medical School, London, UK; 2 Department of Clinical and Movement Neurosciences, UCL Queen Square Institute of Neurology, University College London, London, UK; 3 Neurology Unit, Neuromotor & Rehabilitation Department, Azienda USL- IRCCS di Reggio Emilia, Reggio Emilia, Italy; 4 Department of Brain and Behavioural Sciences, University of Pavia, Pavia, Italy; 5 IRCCS Mondino Foundation, Pavia, Italy; 6 Movement Disorders Center, Neurology Department, Perugia General Hospital, University of Perugia, Perugia, Italy; 7 Department of Nuclear Medicine, Medical School, University of Thessaly, University Hospital of Larissa, Larissa, Greece; 8 Perceptive Discovery, Hammersmith Hospital, London, UK; 9 Faculty of Health, University of Plymouth, Plymouth, PL4 8AA UK; 10 Neuroscience Section, Department of Medicine, Surgery and Dentistry Scuola Medica Salernitana, University of Salerno, Salerno, Italy; 11 School of Medicine, University of Thessaly, Larissa, Greece; 12 Department of Neurology, Queen Elizabeth University Hospital, Institute of Neurological Sciences, Glasgow, UK; 13 Division of Neuroscience, School of Medicine, University of Dundee, Dundee, UK; 14 Medical Research Council Protein Phosphorylation and Ubiquitylation Unit, School of life Sciences, University of Dundee, Dundee, UK; 15 Department of Molecular Medicine, University of Pavia, Pavia, Italy

**Keywords:** Parkin mutation, Parkinson’s disease, Serotonin transporter, PET imaging, Non-motor symptoms

## Abstract

**Purpose:**

We investigated serotonergic and dopaminergic terminal integrity role in *PRKN-*associated Parkinsonism (PRKN-PD) using [¹¹C]DASB Positron Emission Tomography (PET) and [¹²³I]FP-CIT Single Photon Emission Computerised Tomography (SPECT).

**Methods:**

Fourteen PRKN-PD patients (mean age 49.70 ± 10.83, disease duration 10.95 ± 7.59 years, H&Y 2.0 ± 0.68), and twelve iPD patients (mean age 65.40 ± 7.48 years, disease duration 5.05 ± 4.50 years, H&Y 2.0 ± 0.93) underwent clinical assessments, 3-Tesla MRI, [^11^C]DASB PET-CT, and [^123^I]FP-CIT SPECT, and compared with previously acquired healthy control (HCs) data. [^11^C]DASB distribution volume ratio (DVR) parametric images were generated and DVR-1 values, equivalent to BP_ND_, sampled from a priori selected regions-of-interest (ROIs) with the posterior cerebellum as reference. [^123^I]FP-CIT images underwent reconstruction and normalization to standard space, and striatal Specific Binding Ratio (SBR) calculated from the eight hottest consecutive slices.

**Results:**

PRKN-PD patients showed 20.8% to 45.2% [^11^C]DASB BP_ND_ loss across several cortical and subcortical ROIs compared to HCs (*p* ≤ 0.01). After adjusting for age and disease duration, no differences in [^11^C]DASB BP_ND_ were observed between PRKN-PD and iPD. In PRKN-PD, higher [^11^C]DASB BP_ND_ in the raphe, brainstem, ventral striatum, and amygdala, correlated with higher scores on the Non-Motor Symptoms Scale (*p* < 0.05). Higher [^11^C]DASB BP_ND_ in the caudate and putamen correlated with higher scores on the Beck Depression Inventory II scale (*p* < 0.05). No correlation was detected between [^11^C]DASB BP_ND_ and [^123^I]FP-CIT SBR in the caudate and putamen.

**Conclusions:**

PRKN-PD is characterized by widespread serotonergic dysfunction, which is independent of dopaminergic degeneration and linked to key non-motor symptoms, particularly depression. These findings provide novel insights into the pathophysiology of PRKN-PD.

**Supplementary Information:**

The online version contains supplementary material available at 10.1007/s00259-025-07693-2.

## Introduction

Idiopathic Parkinson’s disease (iPD) is characterized by loss of brain serotonergic innervation [1]. *Post-mortem* studies have demonstrated extensive accumulation of Lewy Bodies and Lewy Neurites across the raphe nuclei [2, 3]. The raphe nuclei are the principal source of serotonergic innervation in the brain, projecting extensively to most cortical and subcortical areas [4], and regulating physiological functions such as appetite, sleep, cognition, mood, behaviour, and thermoregulation [1].

In parallel, autoradiography studies have showed that patients with iPD display marked reductions in serotonergic terminal density in several brain regions, including the cerebral cortex, the substantia nigra and the striatum [5, 6]. In vivo studies using Positron Emission Tomography (PET) with [^11^C]DASB, a radioligand with high affinity for the presynaptic serotonin transporter (SERT), have corroborated neuropathological findings. They demonstrated widespread serotonergic terminal loss [7], and revealed correlations with the presence and severity of a wide range of motor and non-motor symptoms, including tremor, levodopa-induced dyskinesias, depression, sleep problems, fatigue, and cognitive impairment [8].

Pathogenic mutations in the *PRKN* gene, encoding for the Parkin protein, represent the most common cause of autosomal recessive monogenic Parkinsonism (PRKN-PD). Clinically, PRKN-PD is characterized by early age of onset, slow progression, a good response to dopaminergic therapy, and a high prevalence of non-motor symptoms [9]. In contrast to iPD, neuropathological examinations of PRKN-PD cases report a relative absence of Lewy pathology and severe neuronal loss in the substantia nigra pars compacta [10]. Nonetheless, the frequent presence of non-motor symptoms such as depression, fatigue, and sleep disorders in PRKN-PD [9] suggests pathophysiological mechanisms affecting serotonergic neurons that are independent of Lewy Pathology.

In physiological conditions, the Parkin protein localizes in several brain regions, including the raphe nuclei [11], where it exerts a protective function against external insults, which may be lost in presence of mutated Parkin [12]. However, the presence and extent of serotonergic pathology in *PRKN* mutation carriers have been rarely investigated in vivo. One prior study in twelve homozygous PRKN-PD, and twelve heterozygous asymptomatic carriers, assessed [^18^F]DOPA binding in extrastriatal regions, to evaluate Aromatic Amino Acid Decarboxylase (AADC) enzyme activity in non-dopaminergic monoaminergic neurons, and found a reduction in the midbrain raphe compared to both healthy controls (HCs) and iPD patients [13]. However, the AADC reflects general monoaminergic, and primarily dopaminergic, function, and is not a specific marker of serotonergic terminal integrity. In contrast, SERT quantification using [¹¹C]DASB is highly specific, given its broad expression throughout serotonergic neurons [14].

The objective of this study was therefore to quantify, in vivo, the integrity of serotonergic terminals in PRKN-PD using PET imaging with the high-affinity tracer [^11^C]DASB, and to compare this with findings in both HCs and patients with iPD. We also aimed to assess, within PRKN-PD participants, the relationship between serotonergic terminal loss in striatal and extrastriatal regions and the severity of motor and non-motor symptoms. Finally, we examined whether serotonergic deficits in the striatum were associated with dopaminergic terminal loss as assessed by [^123^I]FP-CIT SPECT.

## Materials and methods

### Study participants

This study is part of a large, cross-sectional imaging investigation into serotonergic pathology across autosomal dominant and recessive forms of monogenic Parkinsonism. Fifteen symptomatic carriers of pathogenic mutations in the *PRKN* gene and a diagnosis of PD according to the Movement Disorder Society (MDS) clinical diagnostic criteria [15] were referred by a network of specialised centres from the United Kingdom, Italy and Greece and travelled to London to undergo study procedures.

Exclusion criteria included: presence of general contraindications to MRI or PET scanning, current or recent continuous use of medications affecting serotonin transmission (e.g. SSRIs, other antidepressants, triptans), use of medications interfering with [^123^I]FP-CIT SPECT (e.g. neuroleptics, metoclopramide, alpha methyldopa, methylphenidate, reserpine, amphetamine), and history of cancer within five years to consent. One participant was excluded at screening due to the concomitant use of antidepressive medications, resulting in a final PRKN-PD sample size of 14.

The PRKN-PD cohort was compared with 12 iPD patients according to the MDS clinical diagnostic Criteria and no family history or pathogenic mutations, recruited locally. Given the slower disease progression typically seen in PRKN-PD [16], both the PRKN-PD and iPD cohorts were subdivided into ‘early’ and ‘advanced’ subgroups based on disease duration above or below the 50th percentile. This yielded two groups of seven early and seven advanced PRKN-PD patients, and six early and six advanced iPD patients, used for exploratory PET and SPECT analysis. Previously collected [¹¹C]DASB data from 14 healthy subjects recruited as part of previous studies performed at Perceptive London imaging centre and shared as part of this collaboration, served as the control group.

### Clinical procedures

All PRKN-PD and iPD participants underwent comprehensive motor and non-motor symptom evaluations at the NIHR Imperial Clinical Research Facility of Imperial College Healthcare NHS Trust, at Hammersmith Hospital in London. Disease severity and staging was assessed in the OFF state (≥ 12 h off short-acting, ≥ 24 h off long-acting dopaminergic medications) using the MDS-UPDRS [17] and the Hoehn and Yahr scales. Non-motor symptom burden was evaluated using the Non-motor symptoms scale (NMSS); autonomic dysfunction was examined using the Scales for Outcomes in Parkinson’s Disease - Autonomic Dysfunction (SCOPA-AUT) [18]; sleep was assessed with the Parkinson’s Disease Sleep Scale (PDSS) [19]; hyposmia was evaluated using the University of Pennsylvania Smell Identification Test (UPSIT) [20]; presence of depression with the Beck Depression Inventory II (BDI-II) [21]; cognition was screened with the Mini Mental State Examination (MMSE) [22] and the Montreal Cognitive Assessment (MoCA) [23].

To confirm *PRKN* mutations in PRKN-PD and exclude mutations in iPD, venous blood was collected from all participants and analysed with Next Generation Sequencing (NGS) and Multiplex Ligation-dependent Probe Amplification (MLPA) analysis. One PRKN-PD participant had a monoallelic pathogenic mutation (c.823 C > T, p.Arg275Trp). [^11^C]DASB PET and [^123^I]FP-CIT SPECT imaging analysis performed on this participant showed values in line with the mean values of the rest of the PRKN-PD cohort. Main [^11^C]DASB PET and [^123^I]FP-CIT SPECT analysis was repeated including and excluding this subject with no difference in the main results. This subject was therefore retained in the main analysis.

### Scanning procedures

#### PET acquisition

All PET scans were conducted in the OFF state at Perceptive London using either a Siemens Hi-Rez Biograph 6, Biograph 6 TruePoint, or a Siemens Biograph Horizon PET/CT scanner (Siemens Healthcare, Erlangen, Germany). [^11^C]DASB was synthesized according to published methods [24]. Up to 300 MBq of [^11^C]DASB was injected intravenously as a slow bolus over 20 s. Dynamic emission data were acquired continuously for 90 min immediately following injection. Dynamic images were reconstructed into 26 frames (8 × 15 s; 3 × 60 s; 5 × 120 s; 5 × 300 s; 5 × 600 s) using a filtered back projection algorithm (direct inversion Fourier transform) with a 128 matrix, zoom of 2.6 producing images with isotropic voxel size of 2 × 2 × 2 mm^3^ and smoothed with a 5 mm transaxial Gaussian filter.

#### MRI acquisition

T1-weighted MRI scans were acquired for co-registration with the PET images. MRI scans took place either at Perceptive London or at the Mireille Gillings Neuroimaging Centre (MGNC) at the University of Exeter. All MRI scans were acquired on 3-Tesla machines with a 32-channel head coil. MRI scans at Perceptive London were acquired either on a Siemens Magnetom Trio (10 PRKN-PD, nine iPD), or a General Electric (GE Milwaukee, WI, USA) SIGNA hybrid PET-MRI scanner (one PRKN-PD and one iPD). The MRI scans at the MGNC were acquired on a Siemens Prisma (three PRKN-PD and two iPD). Acquisition parameters were as follows for magnetization prepared rapid gradient echo sequence (MPRAGE) on both Siemens scanners: time repetition (TR) = 2300 ms, time echo (TE) = 2.98 ms on the Magnetom Trio or TE = 2.96 ms on the Prisma, flip angle of 9°, time to inversion (TI) = 900 ms, matrix = 240 × 256. An accelerated sagittal inversion recovery fast spoiled gradient echo (IR-FSPGR) was acquired on the GE Signa hybrid PET/MR scanner: TE = 2.99 ms, TI = 400 ms, flip angle of 11°, TR = 6.99 ms.

#### SPECT acquisition

iPD and PRKN-PD participants underwent [^123^I]FP-CIT SPECT scans. SPECT scans took place either on a GE Infinia gamma camera (St Mary’s Hospital, London; eight iPD and 13 PRKN-PD) or on a Siemens Intevo Bold 3-headed gamma camera system (Hammersmith Hospital, London; three iPD). All participants were administered iodine tablets on the scanning morning, an hour or more before tracer injection. Afterwards, participants were given an intravenous injection of up to 185 MBq of [^123^I]FP-CIT, and images were obtained 236.5 ± 12.27 min after injection. Images were acquired in a 128 × 128 matrix, using a 20% energy window and magnification x 2, over approximately 32–40 min. One PRKN-PD could not perform the SPECT scan in London and a recent SPECT scan on a Mediso Anyscan dual head gamma camera (University Hospital of Larissa, Greece) 3.5 h after the administration of 148 MBq of [^123^I]FP-CIT was used for analysis. A Lugol solution was given before the administration for thyroid blockage as part of that scan. One participant of the iPD group dropped out of the study before performing the [^123^I]FP-CIT scan bringing the iPD sample size for [^123^I]FP-CIT SPECT analysis to eleven. To allow comparison with HCs, we downloaded two groups of [^123^I]FP-CIT HC data available from the Parkinson’s Progressive Markers Initiative (PPMI) database. HC [^123^I]FP-CIT SPECT data, and a corresponding T1-weight MRI for registration purposes, used in the preparation of this article was obtained on 19/03/2025 from the PPMI database (www.ppmi-info.org/access-dataspecimens/download-data), RRID: SCR_006431. For up-to-date information on the study, visit www.ppmi-info.org. The two HC groups were matched for age and gender with the PRKN-PD and iPD groups, respectively. Their [^123^I]FP-CIT scans were acquired using a GE Medical Systems SPECT scanner approximately four hours after injection of up to 185 MBq of [^123^I]FP-CIT according to the PPMI protocol procedures.

### MRI analysis

All MR images were reviewed for scanning image quality and artifacts. For each subject, brain extraction, grey matter segmentation, and co-registration was performed using FMRIB Software Library (FSL) “FLIRT” and “FNIRT” functions, or with Statistical Parametric Mapping (SPM, version 12; http://www.fil.ion.ucl.ac.uk/spm/) running on MATLAB (r2018b; the MathWorks, Natick, MA). Regions of Interest (ROIs) were selected a priori as regions with known dense serotonergic innervation and high signal-to-noise ratio on [^11^C]DASB PET images. The frontal cortex, parietal lobe, insular cortex, hippocampus, amygdala, anterior cingulate, posterior cingulate, and brainstem were generated using the Multi-Atlas Propagation with Enhanced Registration (MAPER) [25] applying grey matter masking, except for the brainstem. The hypothalamus, caudate, putamen, ventral striatum, thalamus, dorsal raphe, and ventral raphe were manually delineated, using Analyze medical imaging software (v.14.0 Mayo Foundation AnalyzeDirect), on the subjects’ structural T1-weighted MRI. To assess for any volume differences in PET ROIs between groups, ROI volumes were extracted from manual ROIs Analyze and MAPER using fslstats and normalised to the total intracranial volume.

### [^11^C]DASB PET analysis

[^11^C]DASB PET images were analysed using the Molecular Imaging and Kinetic Analysis Toolbox software package (MIAKAT, version 4.3.15), implemented in MATLAB r2018b. Individual frames were corrected for head motion using a frame-by-frame rigid registration with a reference frame with high signal-to-noise ratio. Quality control was applied on all MIAKAT outputs to ensure all steps of the pipeline were successfully completed. Parametric images of [^11^C]DASB distribution volume ratio (DVR) were generated using the Logan reference method (t*=35 min) [26]. The posterior cerebellar grey matter cortex, excluding the vermis, was manually drawn and used as reference region [27]. [^11^C]DASB DVR images were anatomically co-registered and resliced to the subjects T1 MRI scans using SPM12. ROIs were overlaid on co-registered [^11^C]DASB DVR images. Manual ROIs were sampled in Analyze and MAPER ROIs were sampled using fslstats. The tissue regional binding potential of the specifically bound radioligand relative to the non-displaceable radioligand (BP_ND_) was calculated as DVR-1 [28] and constituted the main outcome measure. All left and right ROIs values were averaged into one single value.

### [^123^I]FP-CIT SPECT analysis

[^123^I]FP-CIT SPECT images underwent reconstruction and attenuation correction. [^123^I]FP-CIT SPECT images were anatomically co-registered and resliced to the subjects T1 MRI scans and subsequently normalized to standard Montreal Neurologic Institute (MNI) space using SPM12. The eight consecutive axial slices with the largest striatal uptake were visually identified and the ROIs, composing of the left and right caudate, and left and right putamen, and the bilateral occipital cortex, used as reference, were manually delineated and sampled using Analyze. The generated values were used to calculate the Specific Binding Ratio (SBR), with the formula: (target region-target reference)/target reference [29]. For analysis between patients and controls, the total caudate and putamen SBR values were used. For comparison between the two groups of PRKN-PD and iPD, and for correlation analysis within the PRKN-PD group, [^123^I]FP-CIT SBR and [^11^C]DASB BP_ND_ values for the caudate and putamen were grouped as clinically most and least affected sides.

### Statistical methods

A preliminary analysis for normality was carried out using the Shapiro-Wilk test. Demographic differences were calculated using parametric or non-parametric tests for continuous variables, where appropriate, and with χ^2^ test for categorical variables. For analysis of MRI ROI volumes, [^11^C]DASB PET BP_ND,_ and [^123^I]FP-CIT SPECT SBR, between-group comparison between controls and patients was performed with parametric or non-parametric tests; between-group comparison between the PRKN-PD and the iPD groups was performed with multivariate analysis of covariance, covariating for age and disease duration, with Bonferroni correction; or non-parametric covariance analysis with Quade’s test, covariating for age and disease duration. Correlation analysis in PRKN-PD between [^11^C]DASB BP_ND_ and clinical characteristics was carried out using Pearson’s correlation analysis, controlling for age where appropriate. Correlation analysis in the PRKN-PD group between [^11^C]DASB BP_ND_ in the caudate and putamen and the [^123^I]FP-CIT SBR in the same areas was carried out using Pearson’s correlation analysis, controlling for age and the time interval between the two scans. Statistical analysis and graph illustration were performed with Statistical Package for Social Science version 28.0 (SPSS, Inc, Chicago, IL, USA), and GraphPad Prism (version 10.0), respectively. Statistical tests are presented as two-tailed, and *p* value threshold was set as < 0.05. All values are reported as mean ± SD, and *p* values as Bonferroni corrected.

## Results

### Demographic and clinical characteristics

A total of 14 PRKN-PD patients, 12 iPD patients and 14 HCs were included in the analysis. Table 1 summarizes the demographic data and clinical characteristics of the three groups. The PRKN-PD group was matched for age with the HC group, but significantly younger than the iPD group. Furthermore, PRKN-PD patients had longer disease duration (*p* = 0.023), and lower scores on the MoCA scale (*p* = 0.013) compared to iPD patients. Conversely, iPD patients scored significantly lower on the UPSIT test compared to the PRKN-PD group (*p* = 0.007).

**Table 1:** Demographic and clinical characteristics of the population studied for [^11^C]DASB analysis

	PRKN-PD (*n* = 14)	iPD (*n* = 12)	HC (*n* = 14)	*p* value PRKN-PD vs. HC	*p* value iPD vs. HC	*p* value PRKN vs. iPD
Age	49.70 ± 10.83	65.40 ± 7.48	44.92 ± 6.31	0.169	**< 0.001**	**< 0.001**
Gender (M: F)	9:5	7:5	14:0	**0.041**	**0.012**	0.536
Disease Duration	10.95 ± 7.59	5.05 ± 4.50				**0.023**
LEDD	312.5 ± 219.76	396.25 ± 375.11				0.486
Hoehn & Yahr	2.0 ± 0.68	2.0 ± 0.93				1.0
MDS-UPDRS I	9.21 ± 4.58	7.00 ± 3.74				0.194
MDS-UPDRS II	7.71 ± 5.80	8.67 ± 6.65				0.703
MDS-UPDRS III	31.36 ± 16.34	27.33 ± 13.56				0.499
MDS-UPDRS IV	4.71 ± 6.29	2.17 ± 3.61				0.212
Total MDS-UPDRS	53.0 ± 26.08	45.17 ± 21.83				0.417
NMSS	49.43 ± 32.97	42.17 ± 29.11				0.556
SCOPA-AUT	15.07 ± 10.49	13.92 ± 6.11				0.731
PDSS	106.23 ± 19.29	120.83 ± 16.66				0.054
UPSIT	29.36 ± 5.18	22.42 ± 6.47				**0.007**
BDI-II	9.36 ± 5.20	7.00 ± 4.41				0.223
MMSE	28.43 ± 1.79	29.17 ± 0.84				0.183
MoCA	27.21 ± 2.16	29.00 ± 1.04				**0.013**

*Abbreviations: BDI-II *Beck Depression Inventory II, *iPD* idiopathic Parkinson’s disease,* LEDD* Levodopa Equivalent Daily Dose, *MDS-UPDRS* Movement Disorders Society Unified Parkinson’s Disease Rating Scale, *MMSE* Mini Mental State Examination, *MoCA* Montreal Cognitive Assessment, *NMSS* Non Motor Symptoms Scale, *PDSS* Parkinson’s Disease Sleep Scale, *PRKN* Parkin mutation carriers, *SCOPA-AUT* Scales for Outcomes in Parkinson’s Disease - Autonomic Dysfunction, *UPSIT* University of Pennsylvania Smell Identification Test. Student’s *t*-test for parametric variables, χ^2^ test

### Region of Interest [^11^C]DASB BP_ND_ analysis in PRKN-PD compared with HCs

ROI-based MRI volumetric analysis, adjusted for age and disease duration, revealed volume reductions in the frontal cortex, temporal lobe, insular cortex and anterior cingulate in PRKN-PD compared to HCs (*p* < 0.05) and in the group of iPD patients compared to HCs in the anterior and posterior cingulate (*p* < 0.05, Supplementary Table 1).

**Fig. 1:**
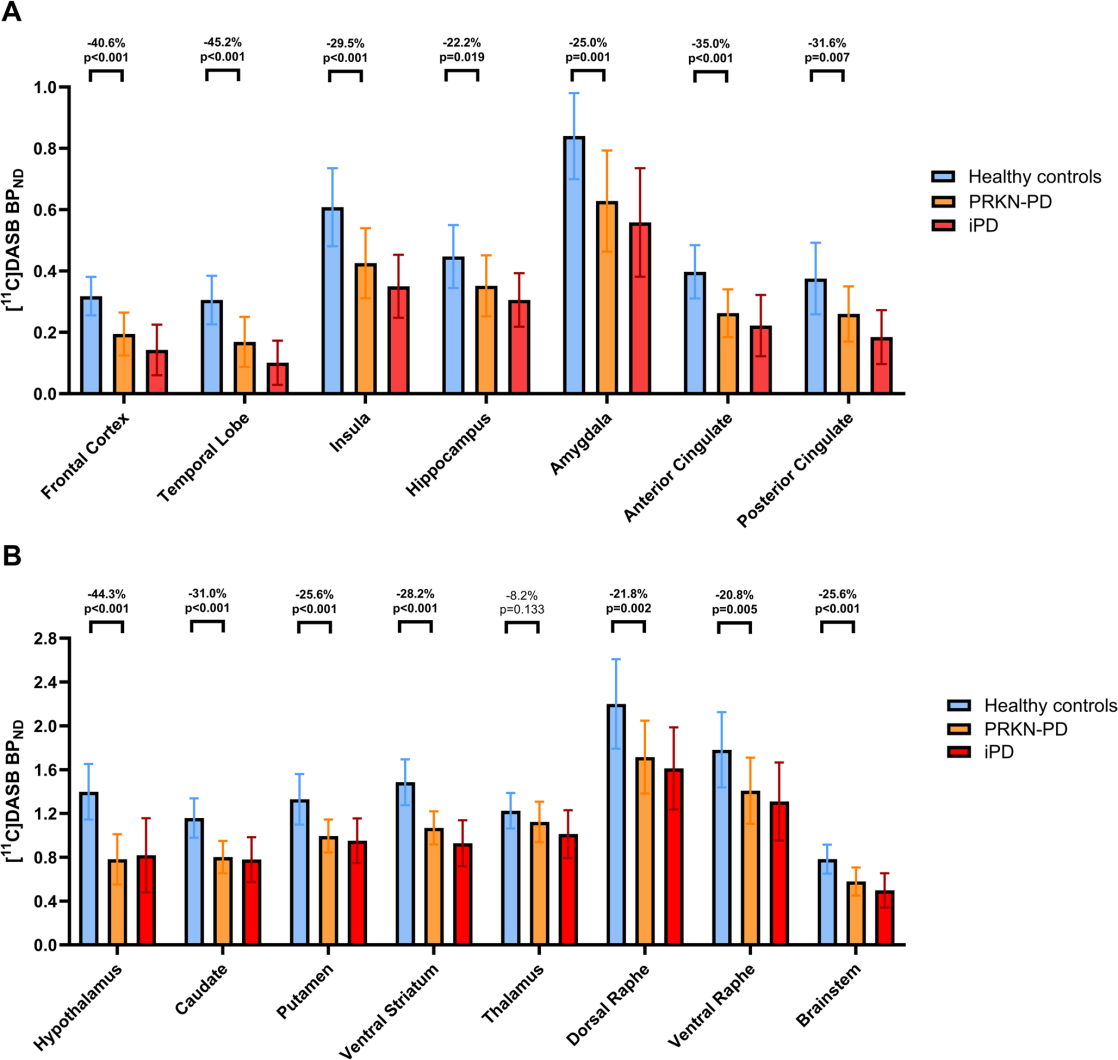
Difference in [^11^C]DASB BP_ND_ between the groups of HCs (in blue), PRKN-PD (in orange), and iPD (in red), across more rostral (**1A**) or more caudal (**1B**) regions of interest. Student’s *t* test for parametric variables, or Mann Whitney *U* test for non-parametric variables; multivariate analysis of covariance between PRKN-PD and iPD with age and disease duration as covariates. Abbreviations: PRKN-PD: *PRKN* carriers with PD; iPD: idiopathic Parkinson’s disease

**Fig. 2:**
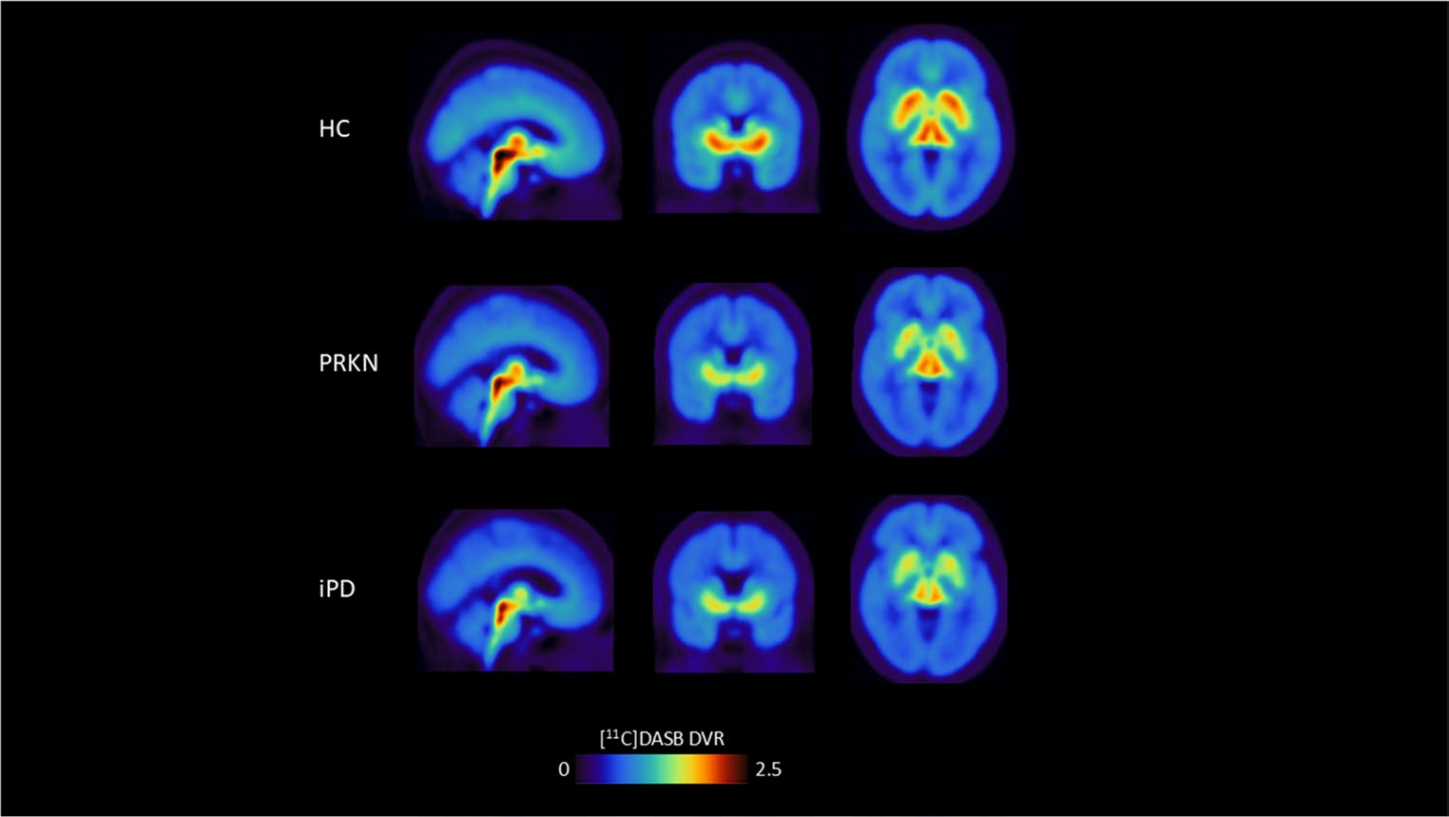
Visual representation of differences in [^11^C]DASB BP_ND_ to the serotonin transporter in the three groups of HCs, PRKN-PD and iPD. Abbreviations: DVR: Distribution Volume Ratio; HC: HCs; iPD: idiopathic Parkinson’s disease; PRKN-PD: *PRKN* carriers with PD

ROI-based analysis of [^11^C]DASB BP_ND_ (Fig. 1, Supplementary Table 2) showed significantly reduced BP_ND_ in PRKN-PD compared to HCs in all cortical and subcortical regions of interest except the thalamus (all *p* < 0.019; thalamus: *p* = 0.133). Percentage reductions in these regions ranged from 20.8% to 45.2%. In cortical areas, the hippocampus showed the smallest change (− 22.2%) and the temporal lobe the largest (− 45.2%). In subcortical areas, losses ranged from − 20.8% (ventral raphe) to − 44.3% (hypothalamus). An illustrative comparison is provided in Fig. 2.

### Region of Interest [^11^C]DASB BP_ND_ in PRKN-PD compared with iPD

Between-group ROI-based comparison on the whole PRKN-PD and iPD groups revealed significant reductions of [^11^C]DASB BP_ND_ in the temporal lobe (−41.2% *p* = 0.033), and in the posterior cingulate (−30.8% *p* = 0.042) in iPD compared to PRKN-PD. After multiparametric analysis, covariating for age and disease duration, these differences did not remain significant (Supplementary Table 2).

We performed exploratory analysis on the subgroups of early and advanced PRKN-PD and iPD groups to evaluate the changes of [^11^C]DASB BP_ND_ according to disease duration. The groups consisted of seven early PRKN-PD (mean age: 41.46 ± 4.73 years; mean disease duration: 5.11 ± 2.62 years) and seven advanced PRKN-PD (mean age: 57.95 ± 8.56 years; mean disease duration: 16.79 ± 6.19 years); and of six early iPD (mean age: 65.24 ± 9.98 years; mean disease duration: 1.25 ± 0.65 years), and six advanced iPD (mean age: 65.56 ± 4.84 years; mean disease duration: 8.86 ± 3.07 years). Full demographic and clinical data available on Supplementary Table 3).

Multivariate analysis of covariance using age and disease duration as covariates, demonstrated significantly lower [^11^C]DASB BP_ND_ in the posterior cingulate in advanced iPD compared with advanced PRKN-PD (*p* = 0.035), with a trend for the temporal lobe (*p* = 0.056, Supplementary Table 4).

### Correlations of [^11^C]DASB BP_ND_ with PRKN-PD clinical characteristics

In the PRKN-PD group, Pearson’s correlation analysis revealed significant correlation between lower [^11^C]DASB BP_ND_ in the frontal cortex, insular cortex, anterior and posterior cingulate, and older age (*p* < 0.05). We performed successive clinical correlation analyses controlling for this parameter.

**Fig. 3:**
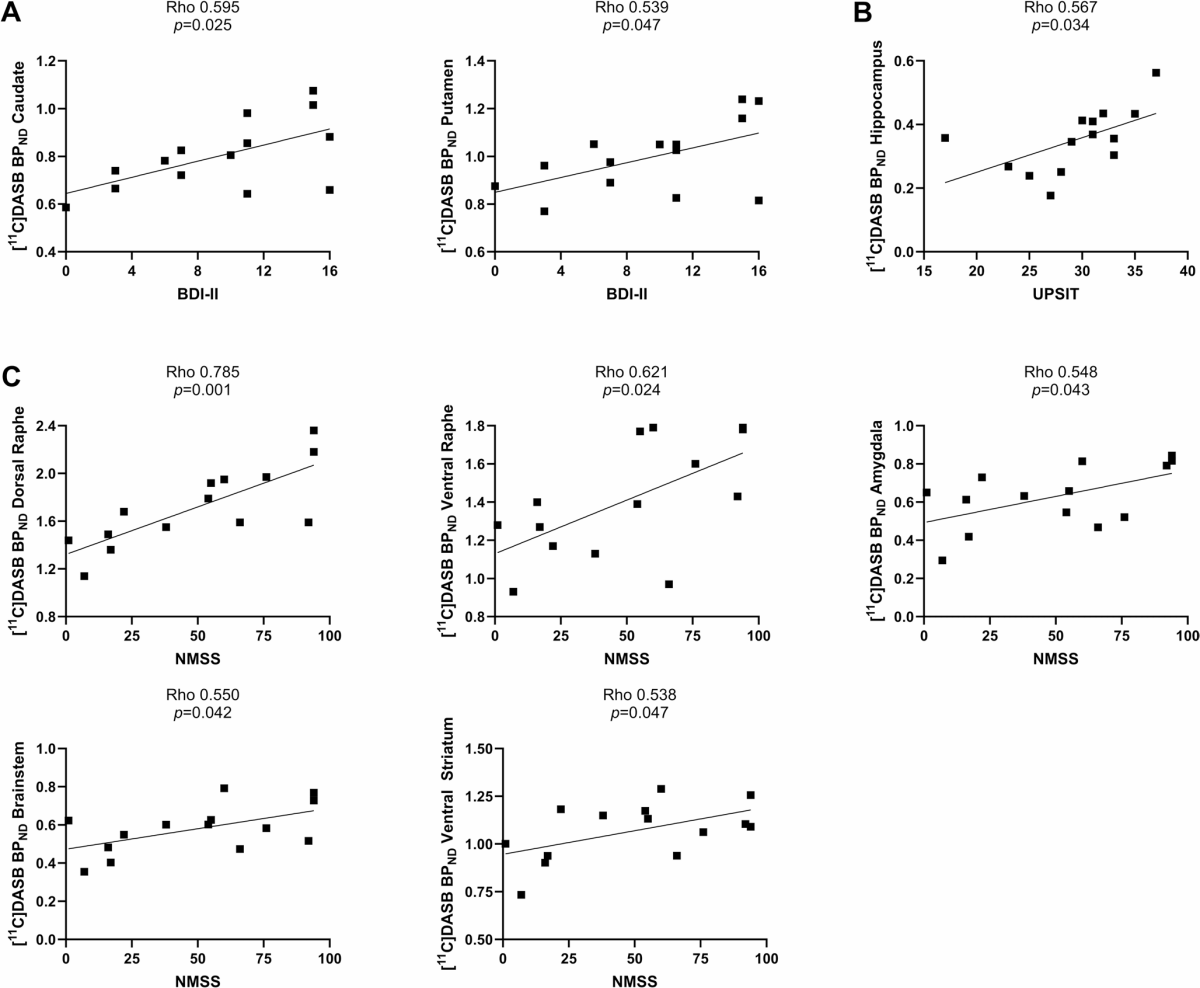
Linear correlation, in PRKN-PD patients, between [^11^C]DASB BP_ND_ to regions of interest and scores on the BDI-II scale (**A**); UPSIT scale (**B**); and the NMSS (**C**). Pearson’s linear correlation analysis. Abbreviations: BDI-II: Beck Depression Inventory II. NMSS: Non motor Symptoms scale. PRKN-PD: *PRKN* carriers with PD. UPSIT: University of Pennsylvania Smell Identification Test

After controlling for age, we identified a significant correlation between higher scores on the BDI-II scale for depression and higher [^11^C]DASB BP_ND_ in the caudate (*r* = 0.595; *p* = 0.025) and putamen (*r* = 0.539; *p* = 0.047) (Fig. 3). When removing the heterozygous carrier, the correlation between BDI-II scores and [^11^C]DASB BP_ND_ remained significant in the caudate (*r* = 0.586, *p* = 0.035). We also identified a correlation between higher scores on the NMSS scale, and higher [^11^C]DASB BP_ND_ in the amygdala, ventral striatum, dorsal and ventral raphe, and brainstem (*p* < 0.05, Fig. 3). Exclusion of the PRKN-PD heterozygous carrier did not affect the significance of the correlation in the dorsal (rho = 0.792, *p* < 0.001) and the ventral raphe (*r* = 0.635, *p* = 0.02). These correlations in the dorsal and ventral raphe also remained significant (*r* = 0.675, *p* = 0.008; and *r* = 0.583, *p* = 0.029, respectively), after re-performing the analysis removing the NMSS mood/cognition subscore.

Additionally, lower [^11^C]DASB BP_ND_ in the hippocampus correlated with lower scores in the UPSIT (*r* = 0.567, *p* = 0.034, Fig. 3). This remained significant after excluding the heterozygous carrier.

### [^123^ I]FP-CIT SPECT in PRKN-PD and iPD groups

Table 2 summarises the demographic and [^123^I]FP-CIT data of the PRKN-PD, iPD and the two HCs groups age- and gender-matched with the two patient groups. Both PRKN-PD and iPD groups showed significantly reduced caudate and putamen SBR compared to HCs (*p* < 0.001). While iPD patients showed slightly lower SBR than PRKN-PD in clinically most/least affected sides, these differences were not significant after adjusting for age and disease duration. No significant correlations were found between [^123^I]FP-CIT SBR and [^11^C]DASB BP_ND_ in the same regions within the PRKN-PD group (**Supplementary Table 5**).

**Table 2:** Age information of the control groups employed for [^123^I]FP-CIT comparison and SBR values across the PRKN-PD, iPD, and the two matched HC groups analysed for [^123^I]FP-CIT SPECT

	PRKN-PD (*n* = 14)	HC PRKN-PD (*n* = 14)	iPD (*n* = 11)	HC iPD (*n* = 12)	*p* value HC vs. PRKN-PD	*p* value HC vs. iPD	*p* value PRKN-PD vs. iPD
Age	49.70 ± 10.83	51.60 ± 10.25	65.40 ± 7.48	65.03 ± 7.00	0.638	0.903	
Gender (M: F)	9:5	9:5	7:4	7:5	1.000	0.794	
Total Caudate SBR	2.17 ± 0.72	3.28 ± 0.77	1.76 ± 0.76	2.85 ± 0.40	**< 0.001**	**< 0.001**	
Total Putamen SBR	1.49 ± 0.48	3.10 ± 0.64	1.26 ± 0.61	2.74 ± 0.47	**< 0.001**	**< 0.001**	
Most affected Caudate SBR	2.11 ± 0.72		1.48 ± 0.84				0.291
Least affected Caudate SBR	2.22 ± 0.73		1.86 ± 0.78				0.256
Most affected Putamen SBR	1.41 ± 0.46		1.20 ± 0.60				0.183
Least affected Putamen SBR	1.56 ± 0.52		1.33 ± 0.64				0.170

*Abbreviations: HC* healthy controls, *iPD* idiopathic Parkinson’s disease, *PRKN* Parkin mutation carriers, *SBR* Striatal Binding Ratio. Comparison for categorical variables performed with χ^2^ test. Between-group comparison between HC and their matched patients group performed with Student’s t-test. Between-group comparison between the PRKN-PD and the iPD groups performed with multivariate analysis of covariance, covariating for age and disease duration

## Discussion

We have employed [¹¹C]DASB PET imaging, which provides high sensitivity for the presynaptic SERT, to quantify serotonergic terminals integrity in patients with PRKN-PD. Compared with HCs, PRKN-PD patients exhibited widespread reductions in [^11^C]DASB binding across multiple cortical and subcortical brain regions, indicating substantial serotonergic terminal loss. Notably, within this overall picture of degeneration, we observed that higher burdens of non-motor symptoms and greater severity of depressive symptoms, measured using the NMSS and the BDI-II scales, respectively, were paradoxically associated with higher [^11^C]DASB binding in several subcortical regions. This counterintuitive finding may reflect an ultimate detrimental effect from homeostatic adjustments taking place in the serotonergic terminals at a synaptic level.

The Parkin protein, encoded by the *PRKN* gene, is an E3 ubiquitin ligase [30] with a central role in promoting mitophagy [31]. Under physiological conditions, wild-type Parkin localizes in the cytoplasma, but under cellular stress, it translocates to damaged mitochondria, increasing their clearance and turnover [32]. By doing this, Parkin reduces the net levels of Reactive Oxygen Species (ROS), achieving a neuroprotective effect. Pathogenic mutations to the *PRKN* gene cause loss-of-function of the Parkin protein, which has the consequence of reducing mitophagy, increasing oxidative stress, impairing respiration, and promoting cell death [33].

In rodent models, Parkin colocalizes with both the Tyrosine Hydroxylase-positive neurons of the substantia nigra, and the serotonin-positive neurons of the raphe nuclei [11]. Although the specific role of Parkin within serotonergic neurons has not been comprehensively studied, there is emerging evidence suggesting that Parkin exerts a protective influence. Rotenone induces serotonergic neurons injury by mitochondrial complex I inhibition and microtubule destabilization [34]. In one study aimed at evaluating the toxicity of Rotenone on serotonergic neurons, the overexpression of wild-type Parkin in these cells attenuated the damage caused by Rotenone to these neurons, whereas the expression of mutated Parkin failed to obtain this neuroprotective effect [12]. Additional support for Parkin’s potentially neuroprotective role comes from studies using 3,4-methylenedioxymethamphetamine (MDMA), whereby *PRKN* knockout rodents exhibited exaggerated hyperthermic responses compared to wild-type animals, despite no significant long-term changes in dopamine or serotonin neurotoxicity markers [35, 36]. Together, these preclinical findings suggest that *PRKN* loss-of-function may heighten the intrinsic vulnerability of serotonergic neurons, possibly contributing to serotonergic terminal loss.

In our study, we observed serotonergic terminal loss in PRKN-PD patients across almost all the cortical and subcortical ROIs examined. This pattern aligns with prior findings in iPD patients, where [^11^C]DASB PET imaging revealed similar patterns of SERT density loss [8]. Compared to iPD, PRKN-PD patients exhibited relatively higher levels of [^11^C]DASB binding in several cortical areas although this difference did not survive covariation for age and disease duration. Previous PET studies in PRKN-PD patients using [^18^F]DOPA reported similar degrees of dopaminergic dysfunction in PRKN-PD and iPD patients [37, 38]. However, PRKN-PD patients notably displayed a slower progression of dopaminergic deterioration compared to iPD [16, 39]. Despite having significantly longer disease duration compared to the iPD cohort, our patients with PRKN-PD displayed similar levels of disease severity, in keeping with the hypothesis of milder clinical progression of their condition [9, 10]. This relative preservation of SERT binding in PRKN-PD may therefore reflect a compensatory or, alternatively, a less advanced disease phenotype. Notably, studies of [^11^C]DASB binding in other monogenic PD forms have revealed variable serotonergic involvement: manifest *LRRK2* mutation carriers showed similar SERT binding to iPD [40] whereas A53T *SNCA* mutation carriers exhibited severe serotonergic loss [41]. These divergent patterns may mirror the known differences in disease severity and progression among monogenic PD subtypes, more severe in *SNCA*, and relatively milder in *PRKN* and *LRRK2*. However, future longitudinal studies, ideally comparing age-matched cohorts, are needed to clarify the progression and clinical significance of serotonergic changes in PRKN-PD.

The SERT protein undergoes dynamic regulatory changes in terminal density to adapt to altered physiological or metabolic demands. This plasticity has been well documented in PET studies on iPD and is thought to underlie the pathophysiological basis of a range of motor and non-motor complications [8]. This mechanism has been evoked in iPD to explain increased [^11^C]DASB binding in the raphe, hypothalamus, caudate, and ventral striatum in response to changes in body mass index [42] and to interpret increased limbic serotonergic innervation in iPD patients with apathy [43]. In our PRKN-PD cohort, correlation analysis controlling for age, revealed that greater non-motor symptom burden was associated with higher [^11^C]DASB binding in the dorsal and ventral raphe nuclei. Additionally, more severe depressive symptoms were associated with higher [^11^C]DASB binding in the caudate and putamen.

With regards to depressive symptoms, two studies have specifically explored its links with serotonergic pathology. Boileau and colleagues reported elevated [¹¹C]DASB binding in the prefrontal and dorsolateral prefrontal cortex in iPD patients with high depression scores [44], while our group found that increased SERT binding in the amygdala, raphe, posterior cingulate, and hypothalamus correlated with depressive symptoms in iPD [45]. Given the raphe nuclei’s central role in serotonergic tone and mood regulation [46], these findings suggest that increased SERT density may represent an insufficient or maladaptive homeostatic mechanism, whereby increased transporter expression actually exacerbates synaptic serotonin depletion by accelerating reuptake. This mechanism would be mirrored in PRKN-PD too. Notably, none of our PRKN-PD participants had a clinical diagnosis of depression or were receiving antidepressant treatment, highlighting that subclinical serotonergic dysregulation may already be biochemically detectable through molecular imaging.

We also investigated the extent of dopaminergic pathology in PRKN-PD by using [^123^I]FP-CIT SPECT, and the relationship between serotonergic and dopaminergic pathology in PRKN-PD patients. We detected similar, severe reduction in [^123^I]FP-CIT binding in PRKN-PD and iPD patients compared to HCs, and we detected no significant correlations between caudate or putamen SERT and DAT availability in the PRKN-PD group. The former finding is consistent with earlier imaging studies comparing PRKN-PD with iPD demonstrating similar, marked, nigrostriatal dopaminergic deficits in PRKN-PD, in the context of its slower clinical progression [38]. The lack of correlation between striatal [^11^C]DASB and [^123^I]FP-CIT, suggests that serotonergic and dopaminergic degeneration may represent distinct and independent pathological processes in PRKN-PD. Serotonergic degeneration may therefore not simply be a downstream marker of dopaminergic neurodegeneration, but may occur through separate mechanisms, particularly in monogenic forms like PRKN-PD.

One subject of our PRKN-PD cohort displayed a heterozygous pathogenic missense mutation (c.823 C >T, p.Arg275Trp). We have performed group analysis on [^11^C]DASB and [^123^I]FP-CIT binding both including and excluding this participant, without detecting any difference in the primary outcome between PRKN-PD and HCs, and in the correlation analysis. There is debate on the biological role of carrying a heterozygous pathogenic mutation to the PRKN gene, and the development of iPD. Recent large studies tend to rule out an increased risk of heterozygous PRKN carriers in developing PD and in its age of onset [47, 48]. At the same time, PET imaging studies on asymptomatic heterozygous *PRKN* mutation carriers have repeatedly showed a significant decrease in [^18^F]DOPA, or [^11^C]CFT in the caudate and putamen compared to HCs [13, 38, 49–51] indicative of an ongoing presynaptic dopaminergic pathology in this population. Future prospective imaging studies targeting asymptomatic heterozygous PRKN mutation carriers could clarify whether early serotonergic alterations co-occur with or precede dopaminergic dysfunction in this genetically defined subgroup.

This study has limitations. First, although we covaried for age and disease duration in between-group analyses, the PRKN-PD and iPD cohorts differed substantially in both variables. These differences limit direct comparisons between groups. Future studies should consider recruiting young-onset iPD patients to better isolate disease-specific effects from age-related changes. Additionally, while our sample size was sufficient for detecting robust between-group effects and correlations, larger cohorts would allow for more granular subgroup analysis (e.g. by genotype or non-motor symptom profile). Another limitation is represented by the use of different PET/CT machines from the same manufacturer to acquire the [^11^C]DASB PET data. However, a comparative analysis of their specifications, including the spatial resolution, yielded perfectly comparable results across the scanners, and the [^11^C]DASB acquisition parameters employed were identical. The design of this study is cross-sectional, and longitudinal imaging studies are needed to understand the temporal trajectory of serotonergic degeneration in PRKN-PD and how it relates to motor and non-motor symptom progression.

In conclusion, this study provides the first in vivo evidence of widespread serotonergic terminal loss in PRKN-PD using [^11^C]DASB PET imaging. These alterations appear to be largely independent of dopaminergic terminal loss, as assessed with [^123^I]FP-CIT SPECT, and are associated with the presence and severity of non-motor symptoms, particularly depression. Our findings suggest that serotonergic dysfunction is a clinically meaningful and biologically distinct feature of PRKN-associated Parkinsonism. Future research should prioritise longitudinal assessments, larger monogenic PD cohorts, and mechanistic studies exploring serotonergic compensatory plasticity and therapeutic modulation. These efforts may ultimately clarify the contribution of serotonergic degeneration to the broader clinical phenotype of PRKN-PD and open new avenues for targeted intervention in non-motor symptoms.

## Supplementary Information

Below is the link to the electronic supplementary material.

Supplementary Material 1

## Data Availability

Data is available upon reasonable request to the Chief Investigator and after signature of a Data Transfer Agreement.

## References

[1] de Natale ER, Wilson H, Politis M. Serotonergic imaging in parkinson’s disease. Prog Brain Res. 2021;261:303–38. 33785134 10.1016/bs.pbr.2020.11.001

[2] Seidel K, Mahlke J, Siswanto S, Krüger R, Heinsen H, Auburger G, et al. The brainstem pathologies of parkinson’s disease and dementia with lewy bodies. Brain Pathol. 2015;25(2):121–35. 24995389 10.1111/bpa.12168 PMC4397912

[3] Halliday GM, Blumbergs PC, Cotton RG, Blessing WW, Geffen LB. Loss of brainstem serotonin- and substance P-containing neurons in parkinson’s disease. Brain Res. 1990;510(1):104–7. 1691042 10.1016/0006-8993(90)90733-r

[4] Di Matteo V, Di Giovanni G, Pierucci M, Esposito E. Serotonin control of central dopaminergic function: focus on in vivo Microdialysis studies. Prog Brain Res. 2008;172:7–44. 18772026 10.1016/S0079-6123(08)00902-3

[5] Kish SJ, Tong J, Hornykiewicz O, Rajput A, Chang LJ, Guttman M, et al. Preferential loss of serotonin markers in caudate versus putamen in parkinson’s disease. Brain. 2008;131(Pt 1):120–31. 17956909 10.1093/brain/awm239

[6] Buddhala C, Loftin SK, Kuley BM, Cairns NJ, Campbell MC, Perlmutter JS, et al. Dopaminergic, serotonergic, and noradrenergic deficits in Parkinson disease. Ann Clin Transl Neurol. 2015;2(10):949–59. 26478895 10.1002/acn3.246 PMC4603378

[7] Politis M, Wu K, Loane C, Kiferle L, Molloy S, Brooks DJ, et al. Staging of serotonergic dysfunction in parkinson’s disease: an in vivo 11 C-DASB PET study. Neurobiol Dis. 2010;40(1):216–21. 20594979 10.1016/j.nbd.2010.05.028

[8] Pagano G, Niccolini F, Fusar-Poli P, Politis M. Serotonin transporter in parkinson’s disease: A meta-analysis of positron emission tomography studies. Ann Neurol. 2017;81(2):171–80. 28019672 10.1002/ana.24859

[9] Menon PJ, Sambin S, Criniere-Boizet B, Courtin T, Tesson C, Casse F, et al. Genotype-phenotype correlation in PRKN-associated parkinson’s disease. NPJ Parkinsons Dis. 2024;10(1):72. 38553467 10.1038/s41531-024-00677-3 PMC10980707

[10] Jerčić KG, Blažeković A, Borovečki S, Borovečki F. Non-motor symptoms of Parkinson`s disease-insights from genetics. J Neural Transm (Vienna). 2024;131(11):1277–84. 39294309 10.1007/s00702-024-02833-8

[11] Horowitz JM, Myers J, Stachowiak MK, Torres G. Identification and distribution of parkin in rat brain. NeuroReport. 1999;10(16):3393–7. 10599851 10.1097/00001756-199911080-00025

[12] Ren Y, Feng J. Rotenone selectively kills serotonergic neurons through a microtubule-dependent mechanism. J Neurochem. 2007;103(1):303–11. 17587308 10.1111/j.1471-4159.2007.04741.x

[13] Pavese N, Moore RY, Scherfler C, Khan NL, Hotton G, Quinn NP, et al. In vivo assessment of brain monoamine systems in parkin gene carriers: a PET study. Exp Neurol. 2010;222(1):120–4. 20043906 10.1016/j.expneurol.2009.12.021

[14] Filip M, Frankowska M, Zaniewska M, Gołda A, Przegaliński E. The serotonergic system and its role in cocaine addiction. Pharmacol Rep. 2005;57(6):685–700. 16382187

[15] Postuma RB, Berg D, Stern M, Poewe W, Olanow CW, Oertel W, et al. MDS clinical diagnostic criteria for parkinson’s disease. Mov Disord. 2015;30(12):1591–601. 26474316 10.1002/mds.26424

[16] Khan NL, Brooks DJ, Pavese N, Sweeney MG, Wood NW, Lees AJ, et al. Progression of nigrostriatal dysfunction in a parkin kindred: an [18F]dopa PET and clinical study. Brain. 2002;125(Pt 10):2248–56. 12244082 10.1093/brain/awf237

[17] Goetz CG, Tilley BC, Shaftman SR, Stebbins GT, Fahn S, Martinez-Martin P, et al. Movement disorder Society-sponsored revision of the unified parkinson’s disease rating scale (MDS-UPDRS): scale presentation and clinimetric testing results. Mov Disord. 2008;23(15):2129–70. 19025984 10.1002/mds.22340

[18] Visser M, Marinus J, Stiggelbout AM, Van Hilten JJ. Assessment of autonomic dysfunction in parkinson’s disease: the SCOPA-AUT. Mov Disord. 2004;19(11):1306–12. 15390007 10.1002/mds.20153

[19] Chaudhuri KR, Pal S, DiMarco A, Whately-Smith C, Bridgman K, Mathew R, et al. The parkinson’s disease sleep scale: a new instrument for assessing sleep and nocturnal disability in parkinson’s disease. J Neurol Neurosurg Psychiatry. 2002;73(6):629–35. 12438461 10.1136/jnnp.73.6.629 PMC1757333

[20] Doty RL, Shaman P, Kimmelman CP, Dann MS. University of Pennsylvania smell identification test: a rapid quantitative olfactory function test for the clinic. Laryngoscope. 1984;94(2 Pt 1):176–8. 6694486 10.1288/00005537-198402000-00004

[21] Beck AT, Steer RA, Brown GK. Manual for the Beck Depression Inventory II. San Antonio TX. Psychological Corporation

[22] Folstein MF, Folstein SE, McHugh PR. Mini-mental state. A practical method for grading the cognitive state of patients for the clinician. J Psychiatr Res. 1975;12(3):189–98. 1202204 10.1016/0022-3956(75)90026-6

[23] Nasreddine ZS, Phillips NA, Bédirian V, Charbonneau S, Whitehead V, Collin I, et al. The Montreal cognitive Assessment, moca: a brief screening tool for mild cognitive impairment. J Am Geriatr Soc. 2005;53(4):695–9. 15817019 10.1111/j.1532-5415.2005.53221.x

[24] Wilson AA, Ginovart N, Schmidt M, Meyer JH, Threlkeld PG, Houle S. Novel radiotracers for imaging the serotonin transporter by positron emission tomography: synthesis, radiosynthesis, and in vitro and ex vivo evaluation of (11)C-labeled 2-(phenylthio)araalkylamines. J Med Chem. 2000;43(16):3103–10. 10956218 10.1021/jm000079i

[25] Heckemann RA, Keihaninejad S, Aljabar P, Rueckert D, Hajnal JV, Hammers A. Improving intersubject image registration using tissue-class information benefits robustness and accuracy of multi-atlas based anatomical segmentation. NeuroImage. 2010;51(1):221–7. 20114079 10.1016/j.neuroimage.2010.01.072

[26] Logan J, Fowler JS, Volkow ND, Wolf AP, Dewey SL, Schlyer DJ, et al. Graphical analysis of reversible radioligand binding from time-activity measurements applied to [N-11 C-methyl]-(-)-cocaine PET studies in human subjects. J Cereb Blood Flow Metab. 1990;10(5):740–7. 2384545 10.1038/jcbfm.1990.127

[27] Kish SJ, Furukawa Y, Chang LJ, Tong J, Ginovart N, Wilson A, et al. Regional distribution of serotonin transporter protein in postmortem human brain: is the cerebellum a SERT-free brain region? Nucl Med Biol. 2005;32(2):123–8. 15721757 10.1016/j.nucmedbio.2004.10.001

[28] Ginovart N, Wilson AA, Meyer JH, Hussey D, Houle S. Positron emission tomography quantification of [(11)C]-DASB binding to the human serotonin transporter: modeling strategies. J Cereb Blood Flow Metab. 2001;21(11):1342–53. 11702049 10.1097/00004647-200111000-00010

[29] Plotkin M, Amthauer H, Klaffke S, Kühn A, Lüdemann L, Arnold G, et al. Combined 123I-FP-CIT and 123I-IBZM SPECT for the diagnosis of parkinsonian syndromes: study on 72 patients. J Neural Transm (Vienna). 2005;112(5):677–92. 15375677 10.1007/s00702-004-0208-x

[30] Shimura H, Hattori N, Kubo S, Mizuno Y, Asakawa S, Minoshima S, et al. Familial Parkinson disease gene product, parkin, is a ubiquitin-protein ligase. Nat Genet. 2000;25(3):302–5. 10888878 10.1038/77060

[31] Hamacher-Brady A, Brady NR. Mitophagy programs: mechanisms and physiological implications of mitochondrial targeting by autophagy. Cell Mol Life Sci. 2016;73(4):775–95. 26611876 10.1007/s00018-015-2087-8 PMC4735260

[32] Narendra D, Tanaka A, Suen DF, Youle RJ. Parkin is recruited selectively to impaired mitochondria and promotes their autophagy. J Cell Biol. 2008;183(5):795–803. 19029340 10.1083/jcb.200809125 PMC2592826

[33] Malpartida AB, Williamson M, Narendra DP, Wade-Martins R, Ryan BJ. Mitochondrial dysfunction and mitophagy in parkinson’s disease: from mechanism to therapy. Trends Biochem Sci. 2021;46(4):329–43. 33323315 10.1016/j.tibs.2020.11.007

[34] Palacino JJ, Sagi D, Goldberg MS, Krauss S, Motz C, Wacker M, et al. Mitochondrial dysfunction and oxidative damage in parkin-deficient mice. J Biol Chem. 2004;279(18):18614–22. 14985362 10.1074/jbc.M401135200

[35] Takamatsu Y, Shiotsuki H, Kasai S, Sato S, Iwamura T, Hattori N, et al. Enhanced hyperthermia induced by MDMA in parkin knockout mice. Curr Neuropharmacol. 2011;9(1):96–9. 21886570 10.2174/157015911795016985 PMC3137210

[36] Sharma A, Bazylianska V, Moszczynska A. Parkin-deficient rats are resistant to neurotoxicity of chronic high-dose methamphetamine. Exp Neurol. 2021;345:113811. 34298012 10.1016/j.expneurol.2021.113811

[37] Ribeiro MJ, Thobois S, Lohmann E, du Montcel ST, Lesage S, Pelissolo A, et al. A Multitracer dopaminergic PET study of young-onset parkinsonian patients with and without parkin gene mutations. J Nucl Med. 2009;50(8):1244–50. 19617340 10.2967/jnumed.109.063529

[38] Hilker R, Klein C, Hedrich K, Ozelius LJ, Vieregge P, Herholz K, et al. The striatal dopaminergic deficit is dependent on the number of mutant alleles in a family with mutations in the parkin gene: evidence for enzymatic parkin function in humans. Neurosci Lett. 2002;323(1):50–4. 11911988 10.1016/s0304-3940(01)02529-0

[39] Pavese N, Khan NL, Scherfler C, Cohen L, Brooks DJ, Wood NW, et al. Nigrostriatal dysfunction in homozygous and heterozygous parkin gene carriers: an 18F-dopa PET progression study. Mov Disord. 2009;24(15):2260–6. 19845000 10.1002/mds.22817

[40] Wile DJ, Agarwal PA, Schulzer M, Mak E, Dinelle K, Shahinfard E, et al. Serotonin and dopamine transporter PET changes in the premotor phase of LRRK2 parkinsonism: cross-sectional studies. Lancet Neurol. 2017;16(5):351–9. 28336296 10.1016/S1474-4422(17)30056-X PMC5477770

[41] Wilson H, Dervenoulas G, Pagano G, Koros C, Yousaf T, Picillo M, et al. Serotonergic pathology and disease burden in the premotor and motor phase of A53T alpha-synuclein parkinsonism: a cross-sectional study. Lancet Neurol. 2019;18(8):748–59. 31229470 10.1016/S1474-4422(19)30140-1

[42] Politis M, Loane C, Wu K, Brooks DJ, Piccini P. Serotonergic mediated body mass index changes in parkinson’s disease. Neurobiol Dis. 2011;43(3):609–15. 21624463 10.1016/j.nbd.2011.05.009

[43] Prange S, Metereau E, Maillet A, Klinger H, Schmitt E, Lhommée E, et al. Limbic serotonergic plasticity contributes to the compensation of apathy in early parkinson’s disease. Mov Disord. 2022;37(6):1211–21. 35238430 10.1002/mds.28971

[44] Boileau I, Warsh JJ, Guttman M, Saint-Cyr JA, McCluskey T, Rusjan P, et al. Elevated serotonin transporter binding in depressed patients with parkinson’s disease: a preliminary PET study with [11 C]DASB. Mov Disord. 2008;23(12):1776–80. 18661545 10.1002/mds.22212

[45] Politis M, Wu K, Loane C, Turkheimer FE, Molloy S, Brooks DJ, et al. Depressive symptoms in PD correlate with higher 5-HTT binding in Raphe and limbic structures. Neurology. 2010;75(21):1920–7. 21098407 10.1212/WNL.0b013e3181feb2ab

[46] Lesch KP, Gutknecht L. Pharmacogenetics of the serotonin transporter. Prog Neuropsychopharmacol Biol Psychiatry. 2005;29(6):1062–73. 15951088 10.1016/j.pnpbp.2005.03.012

[47] Yu E, Rudakou U, Krohn L, Mufti K, Ruskey JA, Asayesh F, et al. Analysis of heterozygous PRKN variants and Copy-Number variations in parkinson’s disease. Mov Disord. 2021;36(1):178–87. 32970363 10.1002/mds.28299

[48] Zhu W, Huang X, Yoon E, Bandres-Ciga S, Blauwendraat C, Billingsley KJ, et al. Heterozygous PRKN mutations are common but do not increase the risk of parkinson’s disease. Brain. 2022;145(6):2077–91. 35640906 10.1093/brain/awab456 PMC9423714

[49] Guo JF, Wang L, He D, Yang QH, Duan ZX, Zhang XW, et al. Clinical features and [11 C]-CFT PET analysis of PARK2, PARK6, PARK7-linked autosomal recessive early onset parkinsonism. Neurol Sci. 2011;32(1):35–40. 20607337 10.1007/s10072-010-0360-z

[50] Khan NL, Scherfler C, Graham E, Bhatia KP, Quinn N, Lees AJ, et al. Dopaminergic dysfunction in unrelated, asymptomatic carriers of a single parkin mutation. Neurology. 2005;64(1):134–6. 15642918 10.1212/01.WNL.0000148725.48740.6D

[51] Binkofski F, Reetz K, Gaser C, Hilker R, Hagenah J, Hedrich K, et al. Morphometric fingerprint of asymptomatic parkin and PINK1 mutation carriers in the basal ganglia. Neurology. 2007;69(9):842–50. 17724286 10.1212/01.wnl.0000267844.72421.6c

