# Supplementary material for: Loss of serotonergic function in carriers of PRKN mutations: a [11C]DASB PET study
Source: Eur J Nucl Med Mol Imaging. 2026 Jan 29;53(6):3960–72. doi: 10.1007/s00259-025-07693-2 (PMC13121179; doi:10.1007/s00259-025-07693-2)
Supplement: Supplementary file 1 — Supplementary Material 1 [file 259_2025_7693_MOESM1_ESM.docx]

**Supplementary material for:**

**Loss of Serotonergic function in carriers of *Parkin* mutations: a [^11^C]DASB PET study.**

| **Contents:** | **Page** |
| --- | --- |
| **Supplementary Figure 1.** Flowchart illustrating the recruitment and study procedures. | 2 |
| **Supplementary Table 1.** Group Comparison of the region of interest volumes. | 2 |
| **Supplementary Table 2.** Differences in [^11^C]DASB BPND across regions of interest between HCs, PRKN-PD and iPD groups. | 3 |
| **Supplementary Table 3**. Demographic and clinical characteristics of the subgroups of PRKN-PD and iPD subdivided by disease duration into early and advanced subgroups. | 5 |
| **Supplementary Table 4.** Comparison of [^11^C]DASB BP_ND_ across regions of interest in the subgroups of PRKN-PD and iPD subdivided by disease duration into early, and advanced subgroups. | 7 |
| **Supplementary Table 5:** Correlations between [^123^I]FP-CIT SBR and [^11^C]DASB BP_ND_ values in the caudate and putamen in the PRKN-PD cohort. | 9 |

**Supplementary Figure 1.** Flowchart illustrating the recruitment and study procedures. Abbreviations: PD=Parkinson’s disease; PPMI=Parkinson’s Progression Marker Initiative; PRKN=Parkin mutation carriers; MRI=Magnetic Resonance Imaging; SPECT=Single Photon Emission Computerised Tomography.

**
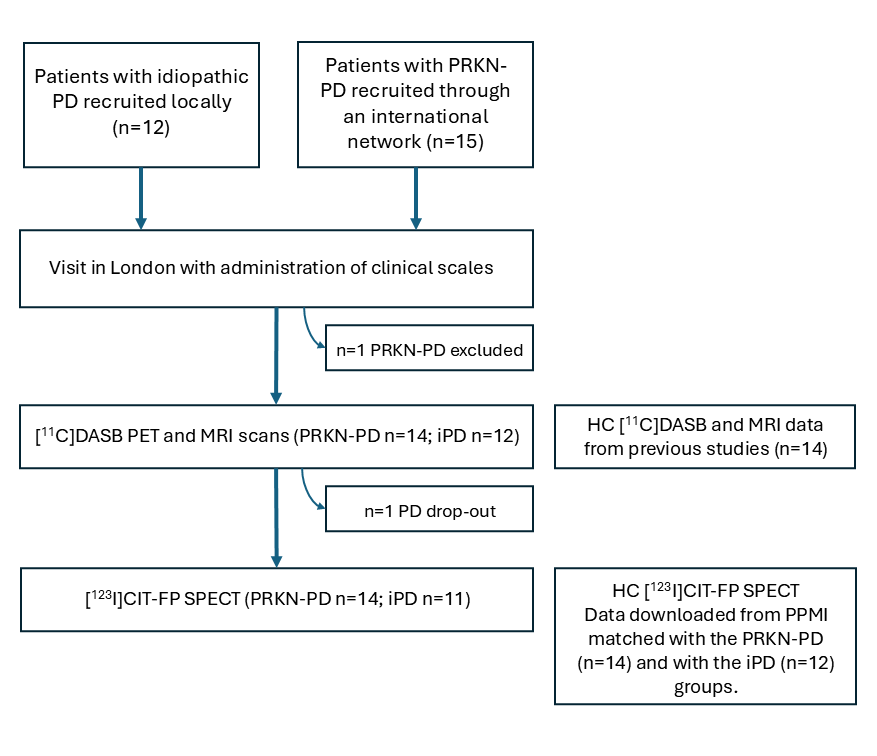
**

| **Supplementary Table 1.** Group Comparison of the region of interest volumes. | | | | | | |
| --- | --- | --- | --- | --- | --- | --- |
|  | **PRKN-PD (n=14)** | **iPD (n=12)** | **HC (n=14)** | ***p* value PRKN-PD vs HC** | ***p* value PRKN-PD vs iPD*** | ***p* value iPD vs HC**** |
| **Frontal cortex** | 3.89 ± 0.43 | 3.82 ± 0.30 | 4.59 ± 0.61 | **0.002** | 0.221 | 0.132 |
| **Temporal Lobe** | 4.00 ± 0.38 | 3.80 ± 0.25 | 4.47 ± 0.52 | **0.012** | 0.564 | 0.05 |
| **Insular cortex** | 0.74 ± 0.07 | 0.74 ± 0.06 | 0.83 ± 0.14 | **0.049** | 0.961 | 0.124 |
| **Hippocampus** | 1.32 ± 0.16 | 1.24 ± 0.13 | 1.45 ± 0.30 | 0.309 | 0.445 | 0.103 |
| **Amygdala** | 0.87 ± 0.12 | 0.87 ± 0.14 | 0.96 ± 0.18 | 0.205 | 0.917 | 0.500 |
| **Anterior Cingulate** | 2.25 ± 0.56 | 2.36 ± 0.34 | 3.07 ± 0.78 | **0.004** | 0.836 | **0.003** |
| **Posterior Cingulate** | 3.11 ± 0.43 | 2.93 ± 0.28 | 3.42 ± 0.46 | 0.086 | 0.815 | **0.024** |
| **Hypothalamus** | 1.07 ± 0.15 | 1.08 ± 0.13 | 1.17 ± 0.28 | 0.266 | 0.762 | 0.930 |
| **Caudate** | 2.18 ± 0.33 | 2.10 ± 0.26 | 2.41 ± 0.59 | 0.211 | 0.109 | 0.592 |
| **Putamen** | 2.83 ± 0.34 | 2.92 ± 0.31 | 3.15 ± 0.60 | 0.087 | **0.015** | 0.969 |
| **Ventral Striatum** | 3.56 ± 0.36 | 3.71 ± 0.53 | 4.01 ± 0.79 | 0.065 | 0.091 | 0.925 |
| **Thalamus** | 2.39 ± 0.24 | 2.39 ± 0.31 | 2.66 ± 0.47 | 0.07 | 0.709 | 0.736 |
| **Dorsal Raphe** | 0.52 ± 0.08 | 0.51 ± 0.05 | 0.57 ± 0.10 | 0.183 | 0.746 | 0.130 |
| **Ventral Raphe** | 0.84 ± 0.18 | 0.81 ± 0.10 | 0.86 ± 0.19 | 0.644 | 0.940 | 0.938 |
| **Brainstem** | 16.40 ± 1.19 | 16.82 ± 1.56 | 17.76 ± 3.12 | 0.139 | 0.209 | 0.364 |
| **Cerebellum Reference region^a^** | 4.40 ± 0.53 | 4.28 ± 0.44 | 4.27 ± 0.53 | 0.526 | 0.263 | 0.174 |
| **Abbreviations:** HC: Healthy controls; iPD: idiopathic Parkinson’s disease; PRKN-PD: PRKN carriers with PD. Between-group comparison between HC and their matched patients group performed with Student’s t-test. *Between-group comparison between the PRKN-PD and the iPD group performed with multivariate analysis of covariance, covariating for age and disease duration. ** Between-group comparison between the iPD group and the HC group performed with multivariate analysis of covariance, covariating for age. ^a^ Manually delineated posterior cerebellar grey matter cortex, excluding the vermis, used as reference region. | | | | | | |

| **Supplementary Table 2.** Differences in [^11^C]DASB BP_ND_ across regions of interest between HCs, PRKN-PD and iPD groups. | | | | | | | | |
| --- | --- | --- | --- | --- | --- | --- | --- | --- |
|  | **PRKN-PD (n=14)** | **iPD (n=12)** | **HC (n=14)** | **% change PRKN-PD vs HC** | **% change iPD vs PRKN-PD** | ***p* value PRKN-PD *vs* HC** | ***p* value PRKN-PD *vs* iPD** | ***p* value PRKN-PD *vs* iPD (covariating for age and disease duration)*** |
| **Frontal Cortex** | 0.19 ± 0.07 | 0.14 ± 0.08 | 0.32 ± 0.06 | -40.6% | -26.3% | **<0.001** | 0.101 | 0.624 |
| **Temporal Lobe** | 0.17 ± 0.08 | 0.10 ± 0.07 | 0.31 ± 0.08 | -45.2% | -41.2% | **<0.001** | **0.033** | 0.156 |
| **Insular Cortex** | 0.43 ± 0.11 | 0.35 ± 0.10 | 0.61 ± 0.13 | -29.5% | -18.6% | **<0.001** | 0.089 | 0.856 |
| **Hippocampus** | 0.35 ± 0.10 | 0.31 ± 0.09 | 0.45 ± 0.10 | -22.2% | -11.4% | **0.019** | 0.222 | 0.869 |
| **Amygdala** | 0.63 ± 0.16 | 0.56 ± 0.18 | 0.84 ± 0.14 | -25.0% | -11.1% | **0.001** | 0.310 | 0.491 |
| **Anterior Cingulate** | 0.26 ± 0.08 | 0.22 ± 0.10 | 0.40 ± 0.09 | -35.0% | -15.3% | **<0.001** | 0.272 | 0.908 |
| **Posterior Cingulate** | 0.26 ± 0.09 | 0.18 ± 0.09 | 0.38 ± 0.12 | -31.6% | -30.8% | **0.005** | **0.042** | 0.258 |
| **Hypothalamus** | 0.78 ± 0.23 | 0.82 ± 0.34 | 1.40 ± 0.25 | -44.3% | 5.1% | **<0.001** | 0.750 | 0.160 |
| **Caudate** | 0.80 ± 0.15 | 0.78 ± 0.20 | 1.16 ± 0.18 | -31.0% | -2.5% | **<0.001** | 0.744 | 0.469 |
| **Putamen** | 0.99 ± 0.15 | 0.95 ± 0.20 | 1.33 ± 0.23 | -25.6% | -4.0% | **<0.001** | 0.551 | 0.409 |
| **Ventral Striatum** | 1.07 ± 0.15 | 0.93 ± 0.21 | 1.49 ± 0.21 | -28.2% | -13.1% | **<0.001** | 0.069 | 0.910 |
| **Thalamus** | 1.12 ± 0.19 | 1.01 ± 0.22 | 1.22 ± 0.16 | -8.2% | -9.8% | 0.133 | 0.076 | 0.819 |
| **Dorsal Raphe** | 1.72 ± 0.33 | 1.61 ± 0.37 | 2.20 ± 0.41 | -21.8% | -6.4% | **0.002** | 0.820 | 0.346 |
| **Ventral Raphe** | 1.41 ± 0.30 | 1.31 ± 0.36 | 1.78 ± 0.34 | -20.8% | -7.1% | **0.005** | 0.462 | 0.294 |
| **Brainstem** | 0.58 ± 0.13 | 0.50 ± 0.16 | 0.78 ± 0.13 | -25.6% | -13.8% | **<0.001** | 0.167 | 0.673 |
| **Abbreviations:** HC: healthy controls; iPD: idiopathic Parkinson’s disease; PRKN: Parkin mutation carriers. Between groups Student’s t test for parametric variables or Mann-Whitney U test for non-parametric variables; *Multivariate analysis of covariance, covariating for age and disease duration, with Bonferroni correction for parametric variables, or Quade’s non-parametric analysis of covariance, covariating for age and disease duration. | | | | | | | | |

| **Supplementary Table 3**. Demographic and clinical characteristics of the subgroups of PRKN-PD and iPD subdivided by disease duration into early and advanced subgroups. | | | | | | |
| --- | --- | --- | --- | --- | --- | --- |
|  | **iPD Early (n=6)** | **PRKN-PD Early (n=7)** | **iPD Advanced (n=6)** | **PRKN-PD Advanced (n=7)** | ***p* value Early iPD vs Early PRKN-PD** | ***p* value Advanced iPD vs Advanced PRKN-PD** |
| **Age** | 65.23 ± 9.98 | 41.46 ± 4.73 | 65.56 ± 4.84 | 57.95 ± 8.56 | **0.001** | 0.073 |
| **Gender (M:F)** | 4:2 | 6:1 | 3:3 | 3:4 | 0.417 | 0.800 |
| **Disease Duration** | 1.25 ± 0.65 | 5.11 ± 2.62 | 8.86 ± 3.07 | 16.79 ± 6.19 | **0.005** | **0.015** |
| **Total LEDD** | 125.00 ± 84.32 | 305.62 ± 245.14 | 667.50 ± 354.76 | 319.38 ± 210.80 | 0.138 | 0.073 |
| **Hoehn & Yahr** | 1.17 ± 0.41 | 1.57 ± 0.53 | 2.83 ± 0.26 | 2.43 ± 0.53 | 0.234 | 0.234 |
| **MDS-UPDRS I** | 7.17 ± 3.92 | 9.14 ± 6.12 | 6.83 ± 3.92 | 9.29 ± 2.81 | 0.498 | 0.234 |
| **MDS-UPDRS II** | 7.17 ± 7.39 | 6.86 ± 2.97 | 10.17 ± 6.11 | 8.57 ± 7.89 | 0.926 | 0.690 |
| **MDS-UPDRS III** | 18.50 ± 9.67 | 26.57 ± 10.43 | 36.17 ± 11.13 | 36.14 ± 20.40 | 0.234 | 0.731 |
| **MDS-UPDRS IV** | 0.00 ± 0.00 | 5.71 ± 7.76 | 4.33 ± 4.18 | 3.71 ± 4.82 | **0.035** | 0.628 |
| **MDS-UPDRS total** | 32.83 ± 19.40 | 48.29 ± 21.88 | 57.50 ± 18.75 | 57.71 ± 30.71 | 0.204 | 0.988 |
| **NMSS** | 37.00 ± 23.29 | 48.43 ± 38.39 | 47.33 ± 35.47 | 50.43 ± 29.64 | 0.525 | 0.869 |
| **SCOPA-AUT** | 11.83 ± 5.04 | 12.86 ± 8.09 | 16.00 ± 6.81 | 17.29 ± 12.71 | 0.787 | 0.822 |
| **PDSS** | 125.17 ± 13.36 | 98.83 ± 14.51 | 116.50 ± 19.67 | 112.57 ± 21.62 | **0.009** | 0.738 |
| **UPSIT** | 25.33 ± 5.32 | 30.43 ± 3.82 | 19.50 ± 6.60 | 28.29 ± 6.40 | 0.083 | **0.034** |
| **BDI-II** | 7.33 ± 3.98 | 7.71 ± 6.21 | 6.67 ± 5.16 | 11.00 ± 3.70 | 0.896 | 0.109 |
| **MMSE** | 29.17 ± 0.98 | 28.57 ± 0.98 | 29.17 ± 0.75 | 28.29 ± 2.43 | 0.299 | 0.392 |
| **MoCA** | 28.83 ± 1.17 | 27.57 ± 2.30 | 29.17 ± 0.98 | 26.86 ± 2.12 | 0.234 | **0.030** |
| **Abbreviations:** BDI-II: Beck Depression Inventory II; iPD: idiopathic Parkinson’s disease LEDD: Levodopa Equivalent Daily Dose; MDS-UPDRS: Movement Disorders Society Unified Parkinson’s Disease Rating Scale; MMSE: Mini Mental State Examination; MoCA: Montreal Cognitive Assessment; NMSS: Non Motor Symptoms Scale; PDSS: Parkinson’s Disease Sleep Scale; PRKN: Parkin mutation carriers; SCOPA-AUT: Scales for Outcomes in Parkinson’s Disease - Autonomic Dysfunction; UPSIT: University of Pennsylvania Smell Identification Test. Student’s t-test and χ^2^ test. | | | | | | |

| **Supplementary Table 4.** Comparison of [^11^C]DASB BP_ND_ across regions of interest in the subgroups of PRKN-PD and iPD subdivided by disease duration into early, and advanced subgroups. | | | | | | |
| --- | --- | --- | --- | --- | --- | --- |
|  | **iPD Early (n=6)** | **PRKN-PD Early (n=7)** | **iPD Advanced (n=6)** | **PRKN-PD Advanced (n=7)** | ***p* value Early iPD *vs* Early PRKN-PD (covariating for age and disease duration)** | ***p* value Advanced iPD *vs* Advanced PRKN-PD (covariating for age and disease duration)** |
| **Frontal Cortex** | 0.19 ± 0.09 | 0.24 ± 0.05 | 0.10 ± 0.06 | 0.15 ± 0.05 | 0.180 | 0.202 |
| **Temporal Lobe** | 0.13 ± 0.08 | 0.21 ± 0.06 | 0.07 ± 0.05 | 0.13 ± 0.08 | 0.655 | *0.056* |
| **Insular Cortex** | 0.39 ± 0.13 | 0.49 ± 0.07 | 0.31 ± 0.05 | 0.36 ± 0.12 | 0.082 | 0.365 |
| **Hippocampus** | 0.31 ± 0.12 | 0.37 ± 0.07 | 0.30 ± 0.05 | 0.34 ± 0.13 | 0.524 | 0.424 |
| **Amygdala** | 0.54 ± 0.24 | 0.68 ± 0.11 | 0.57 ± 0.10 | 0.57 ± 0.20 | 0.118 | 0.646 |
| **Anterior Cingulate** | 0.26 ± 0.13 | 0.29 ± 0.04 | 0.19 ± 0.05 | 0.23 ± 0.10 | 0.096 | 0.163 |
| **Posterior Cingulate** | 0.23 ± 0.10 | 0.30 ± 0.07 | 0.14 ± 0.05 | 0.22 ± 0.09 | 0.332 | **0.035** |
| **Hypothalamus** | 0.83 ± 0.49 | 0.78 ± 0.17 | 0.81 ± 0.11 | 0.78 ± 0.32 | 0.621 | 0.794 |
| **Caudate** | 0.84 ± 0.27 | 0.78 ± 0.14 | 0.71 ± 0.08 | 0.82 ± 0.17 | 0.783 | 0.328 |
| **Putamen** | 1.00 ± 0.28 | 1.00 ± 0.16 | 0.91 ± 0.09 | 0.99 ± 0.15 | 0.744 | 0.666 |
| **Ventral Striatum** | 0.94 ± 0.30 | 1.09 ± 0.12 | 0.91 ± 0.09 | 1.04 ± 0.18 | 0.612 | 0.085 |
| **Thalamus** | 1.00 ± 0.32 | 1.19 ± 0.97 | 1.03 ± 0.06 | 1.06 ± 0.23 | 0.378 | 0.452 |
| **Dorsal Raphe** | 1.47 ± 0.50 | 1.70 ± 0.27 | 1.75 ± 0.12 | 1.73 ± 0.41 | 0.809 | 0.543 |
| **Ventral Raphe** | 1.18 ± 0.46 | 1.40 ± 0.24 | 1.44 ± 0.17 | 1.41 ± 0.38 | 0.624 | 0.936 |
| **Brainstem** | 0.48 ± 0.20 | 0.59 ± 0.09 | 0.51 ± 0.11 | 0.57 ± 0.16 | 0.916 | 0.536 |
| **Abbreviations:** BP_ND_: Non-displaceable binding potential; iPD: Idiopathic Parkinson’s disease; PRKN: Parkin mutation carriers. Multivariate analysis of covariance, with age and disease duration as covariates. | | | | | | |

| **Supplementary Table 5:** Correlations between [^123^I]FP-CIT SBR and [^11^C]DASB BP_ND_ values in the caudate and putamen in the PRKN-PD cohort. | | | | |
| --- | --- | --- | --- | --- |
|  | **Least affected Caudate [^11^C]DASB BP_ND_** |  |  |  |
| **Least affected Caudate [^123^I]FP-CIT SBR** | Rho: 0.350  p=0.291 | **Most affected Caudate [^11^C]DASB BP_ND_** |  |  |
|  | **Most affected Caudate [^123^I]FP-CIT SBR** | Rho: 0.273  p=0.417 | **Least affected Putamen [^11^C]DASB BP_ND_** |  |
|  |  | **Least affected Putamen [^123^I]FP-CIT SBR** | Rho: 0.126 p=0.713 | **Most affected Putamen [^11^C]DASB BP_ND_** |
|  |  |  | **Most affected Putamen [^123^I]FP-CIT SBR** | Rho: -0.023 p=0.947 |
| **Abbreviations:** BP_ND_: Non-displaceable binding potential; PRKN: Parkin mutation carriers; SBR: Striatal binding ratio. Pearson’s linear correlation analysis, controlling for age. | | | | |
